# Ethically governing artificial intelligence in the field of scientific research and innovation^☆^

**DOI:** 10.1016/j.heliyon.2022.e08946

**Published:** 2022-02-16

**Authors:** Elsa González-Esteban y Patrici Calvo

**Affiliations:** Universitat Jaume I, Spain

**Keywords:** Artificial intelligence, Disruptive technologies, Dialogic ethics, Ethical governance, ETHNA System, RRI, Scientific research

## Abstract

Artificial Intelligence (AI) has become a double-edged sword for scientific research. While, on one hand, the incredible potential of AI and the different techniques and technologies for using it make it a product coveted by all scientific research centres and organisations and science funding agencies. On the other, the highly negative impacts that its irresponsible and self-interested use is causing, or could cause, make it a controversial tool, attracting strong criticism from those involved in the different sectors of research. This study aims to delve into the current and virtual uses of AI in scientific research and innovation in order to provide guidelines for developing and implementing a governance system to promote ethical and responsible research and innovation in the field of AI.

## 1 Introduction

Society has traditionally been happy to place a great deal of trust in scientific research, a key element in its emergence and development. As various reports and studies show, society's trust in scientific research has remained high and stable over time. There is currently still an upward trend, which has strengthened in the last decade (ALLEA, 2019 Castell et al., 2014; González-Esteban et al., 2021; FECYT, 2021; Moan et al., 2021; Moan and Ursin, 2021; Strand, 2019). For example, in Spain, 85% of the population would like the national government to devote more resources to scientific research (FECYT, 2021), while in the United Kingdom, 90% of citizens think scientists make a very valuable contribution to society (Castell et al., 2014). And in Europe in general, the vast majority of citizens metaphorically perceive science as a “train on its tracks towards economic growth, increased human welfare and progress” (Strand, 2019).

However, these and other studies also show that scientific research is constantly moving in such complex and uncertain terrain that any economic, political or social change or any disruptive element can cut this trend short and reverse the process, with the negative consequences this would entail for the entire system. Among the main factors to be taken into account to prevent a damaging reversal of the trend are professional malpractice, social dependence on ICTs, and the digital transformation of scientific research.

Firstly, there is concern about the increase in malpractice in the field of scientific research. As shown by various institutions and studies, institutional, economic and cultural factors have encouraged an increase in cases of professional malpractice including fraud, corruption, plagiarism, conflicts of interest, financial doping, improper attribution, illicit appropriation of ideas, concepts and results, influence peddling, falsification of evidence, data manipulation, exaggeration of results, lack of protection of research subjects, misappropriation or misuse of resources, commodification of knowledge, use of phantom sources, nepotistic or inbred citation, improper or fraudulent use of information (Bernuy, 28 November, 2016; Buela-Casal, 2014; Caro-Maldonado, 2019; Salinas, 2005; Sztompka, 2007; Tudela and Aznar, 2013).

In this respect, the website *Retraction Watch. Tracking retractions as a window into the scientific process* (*Retraction Watch*, 2021) highlights between 500 and 600 annual retractions in the field of natural sciences alone “(...) due to the use of unconfirmed or invented data, copies of other works, misuse of statistics, etc.” (González, 2018). Even more worryingly, it points out the recurrence of such behaviour and indifference to it by some researchers. The case of Yoshitaka Fujii, who has the dubious honour of leading the world ranking of retractions, with 183 retracted articles, is a significant one. The ranking of the ten most cited retracted articles also demonstrates that even after retraction they all show a substantial increase in citations (Retraction Watch, 2019).

Secondly, exacerbated dependence on ICTs in the digitally hyperconnected societies of the 21^st^ century is a cause for concern. As the discussion paper “Trust in Science and Changing Landscapes of Communication”, drawn up by All European Academies (ALLEA), points out “(…) the rise of social media and the platformisation of public discourse lead to specific trends that are challenging long-established trustbuilding mechanisms” (ALLEA, 2019: 3). Among the trends encouraged by the digital environment in the field of scientific research are context collapse, confirmation bias and polarisation. These three trends are associated with, and even promoted by, certain economic, political and social phenomena, such as the corporatisation of communication, computational propaganda, political polarisation, and the establishment of new forms of detection and signalling of trustworthiness. The problem with these trends is that: “All of this has substantial consequences for the communication of science and could lead to a pluralisation that might threaten the core pillars of trust in science as well as media: integrity, transparency, autonomy and accountability of researchers and journalists” (ALLEA, 2019: 1).

Finally, the impact of digital transformation on the field of scientific research is worrying. The appearance and application of disciplines such as Artificial Intelligence and its various sub-disciplines (such as machine learning or artificial neural networks), techniques (such as facial recognition or clustering) and design and analysis technologies (such as tensor processing units and *apriori* algorithms*)* in the field of research is now leading to a significant increase in the productive, prospective and predictive capacity of the sector. In this regard, it is making notable contributions in various fields of research. In medical physics and biotechnology, for example, artificially intelligent nanosensors are being used to observe and analyse different biomolecules without compromising their activity, in order to design new treatments for multiple disorders and diseases (John-Herpin et al., 2021). Meanwhile, the universe abounds with huge quantities of data and metadata for physicists and astronomers and the application of Artificial Intelligence is allowing researchers to convert this into relevant, understandable information. This has made it possible to achieve milestones which seemed unattainable until recently, such as the imaging of black holes (Akiyama et al., 2019) and the capture of gravitational waves (Schmitt et al., 2019). In chemistry, machine learning techniques and technologies mean researchers can extract and analyse millions of chemical reactions from the hundreds of thousands of patent documents created over the past 50 years to observe how trends in reactions and the properties of the synthesised products have changed. This has exponentially improved their research because, before this, manual studies had to focus on far fewer reactions (Musib et al., 2017). These are just a few of many cases.^1^

However, the digital transformation of scientific research has also had certain negative impacts on sectors, centres and people involved in research, and also on society, especially on the most vulnerable groups (Calvo, 2021; Prates et al., 2018; Núñ ez Partido, 2019). Firstly, there is a growing smart technology gap affecting researchers and research centres linked to the concentration of Artificial Intelligence in a few regions of the world. This perpetuates, strengthens or generates inequality between sectors, groups and individuals (Soni et al., 2019). Secondly, socially, cases related to the social exclusion of research results based on gender, nationality, religion, among others; the intrusion of AI algorithms into the private and intimate sphere of people under investigation; the enormous deficits of informed consent detected in research processes that use AI techniques and technologies; depersonalisation and shirking responsibility in research processes that apply AI in their development and decision-making; and the negligible or non-existent return for society from its economic and collaborative efforts in designing and developing AI, are among the most important effects in this respect.

The aim of this study is precisely to propose an ethical and responsible governance system for AI for scientific research and innovation centres and science funding agencies. Firstly, a critical analysis of the current or virtual impact of AI on research processes and its main consequences for society, especially affecting the most vulnerable groups, will be carried out. Secondly, the study will critically address the main governmental (from the State) and corporate (from the market and/or civil society) initiatives for the design, development and use of AI in research that lives up to modern digital society's expectations of fairness and accountability. Thirdly, it will put forward a basis for meeting the challenge of ethical and responsible governance of AI in scientific research using the *Responsibility Research and Innovation* (RRI) framework promoted by the European Union, particularly through the SIENNA project. Finally, a design for a governance system will be put forward: the ETHNA System. Developed from the perspective of RRI, this offers scientific research and innovation and science funding agencies the possibility of ethical and responsible governance of the design, development and use of AI.

## 2 Scientific research and innovation in the age of AI

Artificial Intelligence (AI) has become the main disruptive force in 21^st^-century society and its different fields of activity, not always for the better. On one hand, AI offers great opportunities to improve productive, health care, clinical, communicative, participative, decision-making, artistic, research and innovative processes in terms of sustainability, predictability, speed, exhaustiveness, extensibility, capacity, completeness, consistency, efficiency, ratification, precision, detection, entertainment and creativity, among many other things. On the other hand, the use of AI also has a less pleasant side because of its direct or indirect involvement in the exponential increase in the underlying complexity, generating higher levels of uncertainty, inequality, disaffection, instrumentalisation, reification, heteronomy, alienation, anomie and psychopathologies (Calvo, 2020b, 2021; Prunkl et al., 2021).

In the field of scientific research, the potential of AI and its different digital application techniques and technologies, such as machine learning, facial recognition and data mining, has been reflected in a series of developments. These include an exponential increase in scientific productivity; the democratisation of scientific knowledge; the removal of barriers that once limited scientific progress; the possibility of achieving objectives that were unattainable just a decade ago; the increased predictability and control of nature; the development of surprising techniques for observing physical or social phenomena; and the economic, social and environmental sustainability of the scientific research and communication processes.

In this respect there are outstanding paradigmatic cases, such as the publication of the first academic book written by a digital researcher (Beta Writer, 2019); the capture of the first image of a black hole thanks to the use of a massive AI algorithm (Akiyama et al., 2019); and the design of AI chips by AI itself (Norrie et al., 2021). The common denominator of these cases is an AI algorithm powered by big online databases and metadata.

In the field of natural sciences, the cases of Springer's Beta Writer and M87, of the Event Horizon Telescope Collaboration, are particularly outstanding. Beta Writer produced the first academic book written by an Artificial Intelligence algorithm: *Lithium-Ion Batteries: A Machine-Generated Summary of Current Research* (Beta Writer, 2019). This chemistry book, published by Springer Nature, provides an overview of the latest research on lithium-ion batteries. As Christian Chiarcos, head of the project that developed Beta Writer – the first *virtual scientist* – has argued: “This publication has allowed us to show the extent to which the challenges of machine-generated publications can be resolved when scientific publishing experts work with computational linguists” (Pinedo, 10 April, 2019). Meanwhile, M87, developed as part of the Event Horizon Telescope Collaboration project, involved obtaining the first image of a black hole using a massive AI algorithm (Akiyama et al., 2019). This image required eight interconnected telescopes to produce big data and metadata on the M87 black hole, interferometric technology to combine the data and metadata provided by the different telescopes, and an AI algorithm composed of multiple, combined, artificially intelligent mathematical models to convert the data and metadata into relevant information. It shows isomorphism of the event horizon of this massive object, something never before observed by humans.

Elsewhere, in the field of Human and Social Sciences, the *Carabela* and *OpenPose* projects are being developed. *Carabela*, developed in Spain jointly by the Technical University of Valencia and the Centre for Underwater Archaeology of the Andalusian Institute of Historical Heritage (Vidal et al., 2020), managed to show that it is possible to rewrite history using *algorithmic historians*. This worked by applying an AI algorithm to 125,000 digitised documents on Spanish maritime trade and exploration between the 16^th^ and 19^th^ centuries for a few minutes. The process discovered “about 400 references to shipwrecks, half of which we did not have located”. It also found a letter from a Spanish Jesuit dated 10 June, 1710, addressed to King Philip V of Spain, describing “(...) the exact coordinates locating the [southern] continent and its size from east to west”, 80 years before the voyage of James Cook, who had previously been considered to be its Western discoverer (Vidal, quoted by EFE, 8 December 2019). Meanwhile, the *OpenPose* project, developed by the *Visual Recognition Group* at the Czech Technical University, managed to find links and influences previously unknown thanks to an *algorithmic art historian*. After applying a *machine learning* algorithm called OpenPose to large databases of digitised pictorial works, its analysis of the composition or body posture of the characters in the paintings (Jenicek and Chum, 2019) not only found known influences between great artists and works, it also discovered connections never before recognised by art historians. As those responsible for the experiment said, “We experimentally show that explicit human pose matching is superior to standard content-based image retrieval methods on a manually annotated art composition transfer dataset” (Jenicek and Chum, 2019: 1).

Finally, the outstanding achievements in the field of engineering include the development of virtual twins and tensor processing units (TPUs) to improve predictability, optimisation and decision-making. Digital Twins involve creating an artificially intelligent clone of a material or immaterial object or process so it can be subjected to specific stresses, stimuli or events with the aim of predicting its reaction and experimenting with possible solutions and preventive actions. This idea is making its way into fields as diverse as democracy, industry, health, economics and communication to increase political participation, predict anomalies in production processes, forecast illnesses and people's care needs, improve user experiences or increase cognitive bandwidth when dealing with large volumes of information. In this respect, César A. Hidalgo's proposal for *augmented democracy* is worth noting (Calvo, 2020a; Hidalgo et al., 2020; Sáez, 26 June, 2018). He believes it will be possible, by developing *Digitals Twins*, to achieve automated direct participation in political decision-making in the not-too-distant future in order to improve democracy. Meanwhile, TPUs are AI chips developed by Google for application in digital tools like Street View, Google Translate and Google Photos (Norrie et al., 2021). The current development of TPUs has generated one of the most spectacular technological breakthroughs in recent times, as the fourth generation of AI chips has been designed and optimised for the first time by TPUs themselves.

However, despite its underlying potential, digital transformation of the research field is also producing negative impacts linked to the collection, application and use of big data. These are especially serious for the most vulnerable groups in society and various examples can be found. Firstly, cases of malpractice are continually coming to light linked to the breakdown of the limits between public and private spheres (Cinco Días, 6 July 2018; Fang, 18 April 2019; Hern, 26 July 2019). Then there is the black market in big data for scientific use (ECD Confidencial, 10 April 2017; García-Rey, 23 November 2019; Hirschler, 3 March 2018). Thirdly, some individuals and organisations have privileged access to people's private information (Nadal and Victoria, 3 January 2021). Another aspect is the homophobic, xenophobic, aporophobic and misogynist bias detected both in the generation of relevant information and applicable knowledge and in decision-making and its results (EFE, 26 September 2016; Dastin, 10 October 2018; Ferrer, 13 February 2020; Reynolds, 4 October 2017). Meanwhile, all dimensions of inequality are increasing (Arnett, 19 October 2015; Smith, 22 June 2016) and responsibility for actions and decisions involving AI algorithms is being depersonalised and dissolved (Seidel, 2019; Helmore, 17 June 2019; Davey, 26 May 2016). Often there is insufficient anonymisation (the effect or action of unlinking data from the person who generated it) or pseudonymisation (the effect or action of maintaining the confidentiality of data generated by a person) or non-consensual de-anonymisation processes (the effect or action of re-linking data to the person who generated it) (Muñoz, 27 July 2020); Nadal, 3 January 2021; Sanz, 4 June 2017). Informed consent processes in transferring of mass data about individuals linked to a cyber-physical ecosystem are inadequate (ECD Confidential, 10 April 2017; Hidalgo, 7 May 2018; Parris, 23 March 2012). Finally, there are problems of false authorship of research created by AI algorithms (Gehrmann et al., 2019), and the encouragement and tolerance of illegal, anti-social and ethically unacceptable patterns of behaviour and attitudes (Hao, 23 June 2020; O'Neil, 2016).

These and other issues amount to an ethical challenge for scientific research. The solution to it requires a constant critical attitude and appropriate guidelines to avoid increasing the complexity and, consequently, damaging the sustainability of research centres and research funding and the viability and operability of research processes. Above all, there is a danger of exacerbating the vulnerability of the groups and individuals affected by research activity and its results, particularly those belonging to the most fragile groups in society.

## 3 The need for governance of AI

Given the reality-transforming potential of AI and the different techniques and digital technologies involved in its advancement and practical application, over the last five years various public bodies (the State) and private corporations (the Market) have launched regulatory or self-regulatory initiatives for its control and improvement (Hagendorff 2020; Jobin et al., 2019). In this respect, the most important proposals are related to the development of legislative frameworks to govern the design and impacts of AI; codes of ethics, conduct and best practices to guide specific professional practice; ethics committees to address the resolution of conflicts related to the use of AI through dialogue and deliberation; and reports and accountability reports to improve transparency and explainability of the economic, social and environmental impacts of AI.

Government agencies have put forward several proposals for the design, use and impacts of AI with an appropriate legislative framework. In Europe, the *Civil Law Rules on Robotics* (European Parliament, 2017) and the *Artificial Intelligence Act* (European Parliament, 2021) should be highlighted. The *Civil Law Rules on Robotics* (2017) revise and expand the four *Laws of Robotics* enunciated and developed by Isaac Asimov over 40 years (Asimov, 1942, 1950, 1982) to include current expectations such as transparency, confidentiality, and accountability in the development and use of AI. Meanwhile, the *Artificial Intelligence Act* (European Parliament, 2021) is a regulation to be applied in the future by all member states of the European Union with the main aim of ensuring the safe and socially acceptable development and application of AI.

However, these regulatory proposals intended to be transferred to the legislative frameworks of the member countries of the European Union are limited by various problems. For example, the rapid development of AI and its practical application techniques and technologies require constant hurried revision of the legal-political framework to ensure it does not become obsolete and futile. The problem is that, no matter how hard we try to narrow the gap between detecting changes and their impacts and revising the legislative framework, there is always a time lag, with consequences that are difficult to control. Meanwhile, the fact that legislative frameworks are limited to particular countries limits their results and effectiveness in the hyperglobalised processes of digital transformation. As long as there is no international legislation, the possibilities of regulating the design and use of AI are greatly restricted.

The most interesting initiatives from the market or civil society include various proposals for self-regulation based on guidelines and codes of ethics, conduct and best practice for the design, application and use of AI, AI ethics committees and AI impact accountability reports. In the Americas, the United States provides the best example, with more than 20 proposals from different governmental organisations, associations and, above all, private companies on guidelines and codes of ethics, conduct or best practice for the design and use of AI (Jobin et al., 2019). Finally, in Europe, the most important initiatives concern the principles that should serve as guidelines for developing laws, codes, exemplary conduct and best practices, such as the *Ethics Guidelines for Trustworthy AI* (2019), drawn up by the *High-Level Expert Group on Artificial Intelligence*, whose participants were mostly representatives of large private corporations.

However, these self-regulatory initiatives have not escaped both internal and external criticism. A good number of them do not seem to be linked to ethics, but rather to a strategy: avoiding State regulation of AI that limits or prevents the great benefits of its practical application. A paradigmatic example in this respect, and one that has slowed down the implementation of self-regulatory initiatives, has been *Google's Advanced Technology External Advisory Council* (ATEAC). This ethics committee was dissolved only two days after it was set up after leaked details of its members demonstrated that they included people who were avowedly homophobic, xenophobic and misogynistic (Bietti, 2020).

The complicated current relationship between big technology companies and ethicists is also worth noting (Sæ tra et al., 2021; Chomanski, 2021). A clear example was the composition of the *High-level Expert Group* called on by the European Commission to design the *Ethics Guidelines for Trustworthy AI* (2019). As can be seen in the document itself, in the *High-level Expert Group* there is a serious deficit of academics from the field of moral philosophy and an overabundance of big business corporations that develop and/or use Artificial Intelligence. This asymmetry has generated some misgivings and doubts about the true intention of the guidelines. Among other things, there are claims that the guidelines are being instrumentalised by techno-economic interests and large corporations whose only aim seems to be to avoid regulation, or at least to influence it so that it is favourable to them (Cotino, 2017). The decision to eliminate the principle of beneficence (which called for the design and use of AI to be focused on doing good) from the final document (High-level Expert Group, 2019) and the asymmetry of knowledge (too many branches of knowledge linked to STEM and a lack of voices from the field of moral philosophy) and power (overabundance of voices from technology and business corporations) seem to confirm this.

Despite the volume and interest of the various initiatives from government agencies and corporations, there is no doubt that there is still a long way to go to achieve proper control and guidance on the design and application of AI in different fields of activity, such as scientific research using big data. Society's trust in AI is necessary in order to achieve the objectives and expectations associated with it, and legislative frameworks and proposals for self-regulation are plausible ways of generating such trust. However, core problems in the collection, processing and use of big data in science research and funding that weaken such initiatives must be addressed^2^. These include questions over the role of those affected; who decides on the principles and values to define the axiological framework forming the basis from which the rules, actions, decisions and impacts linked to AI are laid down and criticised, and how these decisions are taken; which communication tools allow the effective management of trustworthiness; and how all this can be systemised and applied in a complex field like research centres and scientific research funding agencies.

To this end, ethical frameworks are needed based on a universalist, deontological, proceduralist, hermeneutic, dialogic and critical ethical perspective such as that proposed by Karl Otto Apel and Jürgen Habermas in the 1980s (Apel, 1980; Habermas, 1984, 1987) and developed and marginally expanded, particularly by Adela Cortina, Jesús Conill and Domingo García-Marzá (Cortina, 1986, 1990, 2007, 2010; Cortina et al., 2003; Conill, 2006, 2019; García-Marzá, 1992, 2017, 2019). The aim would be to encourage self-regulation while including existing political and legal regulations. In short, the ethical governance of organisations in which the State, the market and civil society must work together through processes that are as participatory and deliberative as possible (González-Esteban, 2013). In other words, it is necessary to design or redesign governance systems that allow the fair, responsible management of digital practice. These would include, for example, the design and implementation of frameworks that are regulatory – such as laws – or prescriptive – such as guidelines and codes of ethics, conduct and best practice. There should be mechanisms for deliberation and dialogue among those affected by the consequences of the systems, such as ethics committees. Instruments to capture information on the fulfilment of the commitments made, such as ethical lines, would also be needed, together with accountability tools concerning the economic, social and environmental impacts, like explainability reports (García-Marzá, 2017; Calvo, 2020b, 2021; Calvo and Egea-Moreno, 2021).

There are now some interesting proposals for governance systems which to some extent attempt to absorb and resolve the problems underlying the development, application and use of AI. This is the context for the ethical challenges in the creation, dissemination and use of information in the big data era, as has already been mentioned. Several initiatives promoted by the European Union are particularly important in this respect. Firstly, there is the proposal for governance in the *AI Watch Artificial Intelligence in Public Services Overview of The Use and Impact of AI in Public Services in the EU* (Misuraca and van Noordt, 2020). This is limited to the field of public administration. It considers the issue as a dilemma and offers an ethically insufficient concept of “governance 'with and of' AI”. Meanwhile, in its Title VI, the aforementioned *Artificial Intelligence Act* (European Parliament, 2021) establishes a clearly insufficient governance system based merely on the establishment of a European Artificial Intelligence Committee to ensure compliance with the implementation and enforcement of the regulations and encourage the exchange of best practices. The *Data Governance Act* (European Parliament, 2020) does suggest certain technical instruments to ensure the preservation of protection, privacy and confidentiality in the transfer, reuse and recovery of data by third parties. However, the horizontal governance it proposes is concerned only with compliance with current legislation, deliberately excluding those affected from the entire participatory process. Finally, for the case we are dealing with in this study, the proposal by *Responsibility Research and Innovation* (RRI) (European Commission, 2012), is based on a problematic perspective of moral conflict and a concept of “science with and for society”, which is very concerned with the inclusion and participation of those affected by the assessment, development and decision-making processes related to scientific research (European Commission, 2012). The steps being taken as part of this proposal are shown below, along with the tangible results being achieved linked to the development of AI in scientific research and innovation organisations, whether they are science funders or science producers.

## 4 Responsible research and innovation for AI: organisational commitment

A framework for responsible research and innovation (RRI) points to the need to ensure that both the results and the design and development processes in research live up to societal interests and ethical expectations. This concept of responsibility insists that “Ensuring ethically aligned AI systems requires more than designing systems whose result can be trusted” (Dignum, 2019, p. 2), as it is necessary to ask how they are designed, why they are designed and who is involved in such designs. These are questions to be stimulated by science and innovation funding agencies and centres and answered within laboratories and research centres. The central aspect involves bearing in mind, following the thesis maintain by the mathematician Nobert Wiener in 1960 and subsequently taken up by Russell, that “[W]e had better be quite sure that the purpose put into the machine is the purpose which we really desire” (Russell, 2016: 58). A fundamental key is clearly defining the “we” and how this “we” is takes effect in the design and development processes and results of research and innovation, as well as in AI funding processes. The answer will be complex because in many cases there is shared responsibility distributed over multiple, interrelated moments. It is therefore necessary to design ambitious forms of AI governance that encourage the chain of responsibility involving all the actors and recognise the overlaps and interrelationships.

An important advance within this framework is offered by the recently completed European research project SIENNA, which for five years (2017–2021) has worked to develop ethical frameworks, operational guidelines for ethics committees, codes of conduct and recommendations for the development of new technologies in accordance with socio-economic and human rights standards. Artificial intelligence and robotics have formed one of the three technological areas covered by this project, along with human enhancement and human genomics. The objective driving the teams involved in this European project has been to promote responsible AI and robotics in line with what society considers to be desirable and ethically acceptable.

Firstly, SIENNA reveals that although there are different international guidelines and recommendations focusing on AI and robotics, not all of them consider research and innovation processes as such. The project's main task, therefore, has been to ask whether such guidelines can be used to orientate research and innovation processes and to interpret their translation to these contexts through participatory consultations with key stakeholders in AI and robotics research and innovation.

The result has been a proposed adaptation of the guidelines developed by the High-Level Expert Group on Artificial Intelligence (2019) for research and innovation centres. This adaptation includes six principles: human agency; privacy and data governance; transparency; fairness; individual, social and environmental well-being; and accountability and oversight. The principle of technical robustness and safety has not been contemplated and a recommendation on design ethics has been added.

The six principles, plus the general recommendation, should provide the minimum content that a morally pluralistic society expects from research and innovation processes in the field of AI and its technological application in society.

*Human agency* includes aspects concerning autonomy, dignity and freedom. It promotes the development of AI and its applications where humans are in control of the systems as much as possible. Thus, the AI must be designed in such a way that: (a) decisions that are personal, political or concern the community are not left in its hands, particularly those concerning individual rights or economic and social matters; (b) basic freedoms are not eliminated or restricted; (c) it does not subordinate, coerce, deceive, manipulate or dehumanise people; and (d) it does not stimulate dependency or addiction.

*Privacy and data governance* points out that all AI must respect the right to privacy. Therefore, the AI's use of data must be actively governed, i.e., monitored and modified if necessary. For this reason, adherence to GDPR (General Data Protection Regulations) is encouraged, as well as the use of human auditing processes for data use.

*Transparency* will enable human agency and data governance, accountability, oversight and human governance to be exercised. It is therefore supremely important to try to ensure that humans can understand how AI works and how AI decisions are made. This principle applies to all elements of AI: data, functionality, and the design, deployment and operational processes. A good example of this is XAI; eXplicable AI. It is also critical that it is always clear to end users who or what they are interacting with – whether it is a person or an AI system, e.g. a chatbot. Another feature of transparency has to do with open communication, which involves disclosing the purpose of the AI, the capabilities and limitations that have been analysed, the foreseeable benefits and risks and the decisions made using AI, as well as the governance processes. In particular, there is a strong need for the design of accountability, as well as keeping records of all decisions on ethical issues made during the AI design and construction process.

*Equity* requires, first of all, that AI be developed and implemented in such a way that all people can have the same rights and opportunities and no-one is undeservedly favoured or disadvantaged. Ensuring compliance with this principle of fairness involves avoiding algorithmic bias in input data, modelling and algorithm design. Algorithmic bias is a specific concern that requires specific mitigation techniques. Applications should specify: (i) how to ensure that data about individuals is representative and reflects their diversity; (ii) how errors in the input data will be prevented; and (iii) how the design of the algorithm will be checked to ensure it does not target certain groups of people unfairly. Secondly, equity also implies AI designed and engineered for universal accessibility. AI systems should be designed so they can be used by different types of end user with different capabilities. Applications should explain how this will be achieved, for example, through compliance with relevant accessibility guidelines. Finally, equity is linked to the search for fair impacts, which requires (i) evidence that potential negative social impacts on certain groups (e.g., the impact on work) have been taken into account and (ii) specification of the measures that will be taken to ensure the system does not discriminate or cause others to do so.

With *individual, social and environmental well-being* we promote research and innovation in AI that does no harm and seeks the well-being of all stakeholders. Where potential risks are identified, measures should be put in place to help mitigate potential negative impacts in the future.

*Accountability and supervision* make it possible to track the agent of responsibility for AI developments and applications at all times, even if the agent is a multiple actor. Those who have built or use or apply the AI must be accountable. They are responsible for the actions generated or the effects the AI produces. From this principle it can be deduced that: (i) developers must be able to explain how and why a system acts as it does, while the supervision of the AI system must also be specified unless compelling reasons are provided to show that it is not necessary. (ii) Applications must explain how undesirable effects will be detected, stopped and avoided. (iii) Where necessary there must be a formal ethical risk assessment. To ensure that the AI complies with the principle of supervision, it must be possible for it to be understood as well as supervised. Its design and operation must be controlled by a human being. There must also be documented procedures for risk assessment and mitigation, as well as a system to ensure the different stakeholders can report their concerns. Finally, all AI systems should be auditable by independent third parties: including the development process by which it was created. The audit must look not only at what was done, but why.

These six principles are complemented from the SIENNA project with a recommendation on Ethics by Design in this sense establishing the need to proactively integrate ethical consideration in the design process and to add special guidelines for systems, data and applications. For example, AI applications in medicine, politics, economics and the workplace; subliminal or addictive AI; ethically aware, autonomous or semi-autonomous AI; processing of sensitive data, predictive analytics in relation to people and their behaviours and their use in educational, work or policy-making domains and so on.

The process of identifying and defining these six principles and the recommendation for ethically acceptable AI and robotics requires organisations that not only recognise such guidelines but also generate a culture around them.

## 5 Ethical governance of AI research and innovation: the ETHNA system as an organisational commitment

One way to achieve this recognition and generation of a culture of responsible AI research and innovation is the institutional design of innovation and research centres that recognise and manage their organisational commitment to ethical and responsible AI. This makes it capable of organisationally addressing some of the ethical challenges outlined above regarding the creation, dissemination and use of information in the era of Big Data and Artificial Intelligence.

It is a governance system that recognises regulatory frameworks but goes beyond them by generating self-regulation in conjunction with the market and civil society. In short, governance in which the State, the market and civil society work together on the ethical guidance of AI research and innovation. This self-regulation affects both the organisations that fund science and those that develop it.

Along these lines, the ETHNA System project proposes a system of ethical governance of research and innovation processes, both in research performance organisations (RPOs) and in organisations that fund research and innovation (research funding organisations). The aim is to help them, on one hand, to consider the consequences of their activities and, on the other, incorporate ethical expectations into their work. The ETHNA System is thus intended to promote structures and processes for the ethical governance of research and innovation in any field (González-Esteban, 2019; Owen et al., 2013), including AI.

The ETHNA System consists of a structure that serves as the basis for the system called RRI Office(r), which allows the alignment of the existing resources and structures in the organisation linked to the key dimensions and areas of responsible research and innovation. It identifies the commitment to responsible research and innovation the organisation wants to take on, establishing an action plan that takes the existing resources and moves steadily towards achieving a committed organisation driving ethically responsible research and innovation.

In order to develop its action plan and achieve its commitment, this RRI Office(r) relies on a Code of Ethics and Best Practices, a Research and Innovation Ethics Committee and an Ethics Line. All these governance structures are monitored with indicators of system progress and performance.

**Figure:**
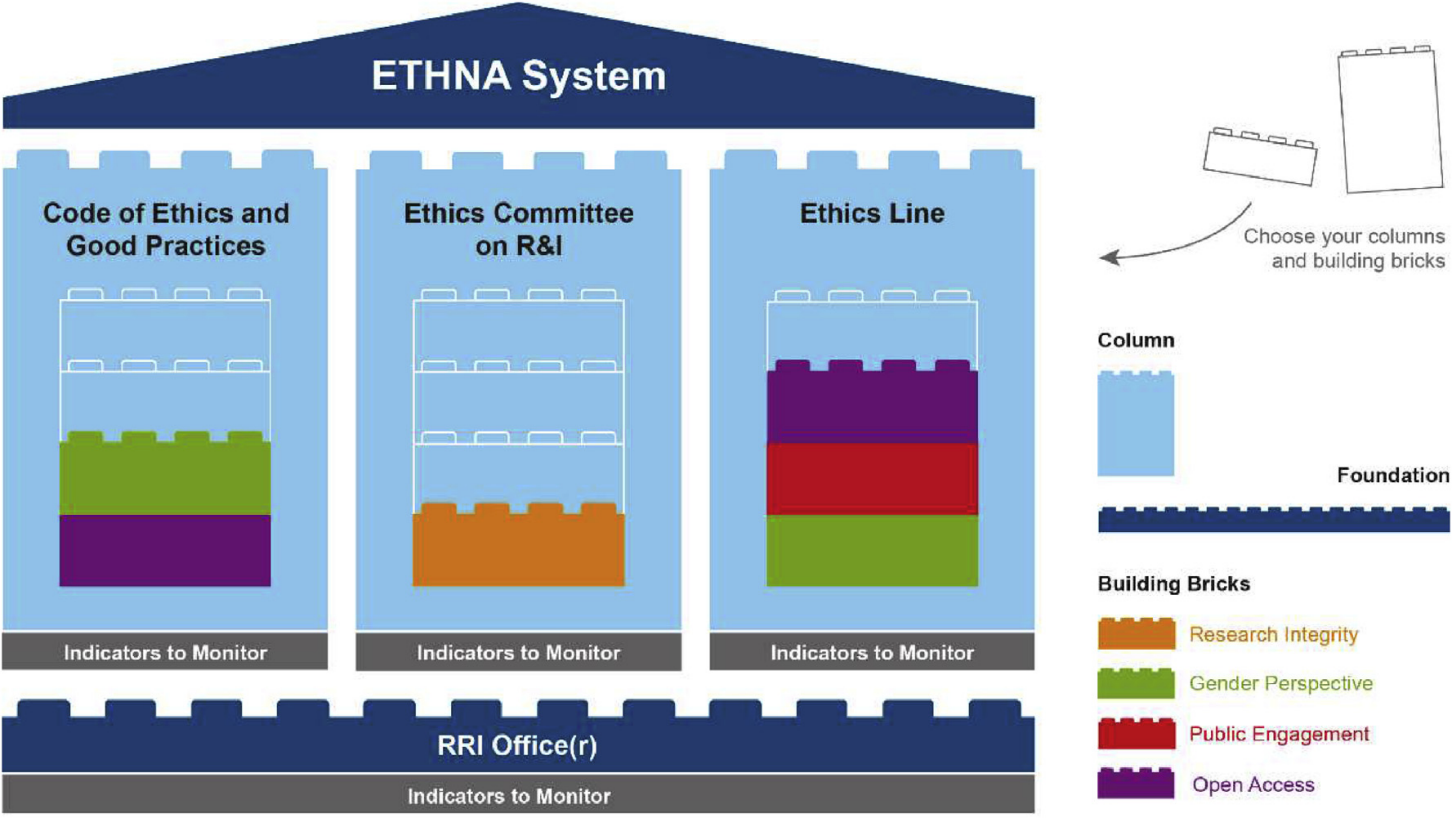
Source: González-Esteban et al. (2021).

The dimensions promoted by this structure are: anticipation, inclusion of all stakeholders linked to the research activity, consideration of the design, development and impact of research and innovation, and the internal and external accountability every institution should have. The areas covered by the ETHNA System are: governance, research integrity, gender perspective, public engagement and open access.

The most outstanding feature of the ETHNA System is that it offers a flexible ethical governance system from which each institution can select and use the parts it needs (González-Esteban et al., 2021). This applies whether the institution is an RFO or RPO and whatever the context it operates in: for example, universities, technology parks, innovation centres, applied research and technology centres, etc.

An organisation engaged in AI research or innovation or an organisation funding AI-related projects or businesses might want to adopt its ethical system of governance to make a commitment to socially desirable and ethically acceptable AI. Specifically, we can speak of ten arguments that could support such a commitment to adopting the ETHNA System of ethical governance by organisations linked to AI research and innovation:

1. To generate credibility and reliability (trustworthiness) in the activity and results achieved by the organisation in R&I.
2. To align the organisation's policies and strategies with European guidelines and thus increase the possibilities for cooperation and funding.
3. To facilitate stable relationships with stakeholders by including them in participatory spaces so their legitimate interests are considered and, as a result, the quality of results improved.
4. To promote a culture that fosters cohesion and a common decision-making position, as well as a healthy working environment that inspires confidence.
5. To encourage a proactive position towards the current challenges of R&I: integrity, gender perspective, public engagement, and open access.
6. To involve stakeholders to increase economic profitability with the rational and

sustainable use of scarce resources.

7. To reduce internal and external coordination costs deriving from possible conflicts and misconducts that have an economic and reputational impact.
8. To position the organisation in terms of RRI by building trust and a reputation for excellence in R&I.
9. To build the character of the organisation by promoting or complying with various existing political and legal frameworks.
10. To promote a close relationship with the community and its needs by responding to the expectations of society (e.g., sustainability, social justice, gender perspective, and integrity research, etc.).

Above all, it should be stressed that the use of this system would increase the credibility and confidence of society and policy makers in AI-based research and innovation.

This is firstly because, from the Code of Ethics and Best Practice, the users and/or customers who receive research results, prototypes and advances would know the values and standards that have been followed in the design, development and/or sale and marketing of AI-based services or products. They would therefore have a guarantee that internal and external stakeholders have been involved and have carefully considered the values and social and ethical standards that have guided the research and innovation. Secondly, as the Code of Ethics and Best Practice that guides such research and innovation is linked to the Ethics Line, any stakeholder (e.g. a potential investor, a local authority or an employee) can ask the company about the way privacy and consent are guaranteed, for example when using “loneliness bracelets for elderly people" or "recognition drones for private use", or inquire about the information base that has been used to develop an algorithm. Thirdly, there is a guarantee that the organisation is accountable, because it has to provide a response via the RRI Office and performance indicators. The manager of the RRI office would therefore receive inquiries, suggestions, reports or complaints and analyse them internally in light of the code adopted. If necessary, they would take the matter to the Ethics Committee on R&I for consideration and deliberation.

As can be seen, the key lies in generating internal reflection on the values and standards that guide the research and innovation process. Participation and deliberation together with civil society and experts in the various critical areas of RRI – the gender perspective, citizen science, integrity and open access – are crucial.

As experience has already shown, non-participatory Codes of Ethics that are not used for decision making and Ethics Committees of external experts making complacent, general recommendations for the organisation do not amount to the institutionalisation of ethics in AI research and innovation (Chomanski, 2021; Yeung et al., 2020; Greene et al., 2019). Ethics should generate an internal culture and identity based on permanent, joint reflection with internal and external stakeholders on the values that should guide research and innovation. These guidelines are then translated into behaviours and procedures based on best practice. The outcome of the research and innovation, as well as the design and process leading to it, must also be open to scrutiny and knowledge. This generates trust in AI as a result of discursively responsible research and innovation.

There are many different ethical challenges in the creation, dissemination and use of information in the era of Big Data and Artificial Intelligence. They change very quickly and often unpredictably.

As this study has shown, one of the most pressing challenges identified, calling for a structural response, involves building systems of ethical governance for research and innovation that enable organisations to self-regulate within existing regulatory frameworks, sometimes going beyond the aims of the latter. However, these self-regulatory frameworks must be established by the State, the market and civil society working together to jointly define the principles that should govern AI research and innovation activity so that it is socially acceptable and ethically desirable. The proposed ETHNA system of ethical governance could be a good way to institutionalise the principles of human agency, privacy and data governance, transparency, fairness, individual, social and environmental well-being, accountability and supervision, as well as the recommendation for Ethics by Design in the era of Big Data and AI.

## Declarations

### Author contribution statement

Elsa Gonzalez Esteban & Patrici Calvo: Conceived and designed the experiments; Performed the experiments; Analyzed and interpreted the data; Contributed reagents, materials, analysis tools or data; Wrote the paper.

### Funding statement

This work was supported by Scientific Research and Technological Development Project “Applied Ethics and Reliability for Artificial Intelligence” PID 2019 109078RB-C21 funded by MCIN/AEI/10.13039/501100011033, as well as in the development of the European Project “Ethical governance system for RRI in higher education, funding and research centers” [872360] funded by the Horizon 2020 program of the European Commission.

### Data availability statement

No data was used for the research described in the article.

### Declaration of interests statement

The authors declare no conflict of interest.

### Additional information

No additional information is available for this paper.

## Footnotes

1 For further insight into the contributions of Artificial Intelligence to the field of scientific research, see Musib et al.; Miller (2019); Hermann and Hermann (2021); Hogarty et al. (2019); Soni et al. (2019); Munim et al., 2020; Vollmer et al. (2018).

2 For a better understanding of such limitations, see d'Aquin et al. (2018).
